# Baseline Splenic Volume Outweighs Immuno-Modulated Size Changes with Regard to Survival Outcome in Patients with Hepatocellular Carcinoma under Immunotherapy

**DOI:** 10.3390/cancers14153574

**Published:** 2022-07-22

**Authors:** Lukas Müller, Simon Johannes Gairing, Roman Kloeckner, Friedrich Foerster, Arndt Weinmann, Jens Mittler, Fabian Stoehr, Tilman Emrich, Christoph Düber, Peter Robert Galle, Felix Hahn

**Affiliations:** 1 Department of Diagnostic and Interventional Radiology, University Medical Center of the Johannes Gutenberg University Mainz, 55131 Mainz, Germany; lukas.mueller@unimedizin-mainz.de (L.M.); fabian.stoehr@unimedizin-mainz.de (F.S.); tilman.emrich@unimedizin-mainz.de (T.E.); christoph.dueber@unimedizin-mainz.de (C.D.); 2 Department of Internal Medicine I, University Medical Center of the Johannes Gutenberg University Mainz, 55131 Mainz, Germany; simonjohannes.gairing@unimedizin-mainz.de (S.J.G.); friedrich.foerster@unimedizin-mainz.de (F.F.); arndt.weinmann@unimedizin-mainz.de (A.W.); peter.galle@unimedizin-mainz.de (P.R.G.); 3 Department of Interventional Radiology, University Hospital Schleswig-Holstein–Campus Luebeck, 23562 Luebeck, Germany; roman.kloeckner@uksh.de; 4 Department of General, Visceral and Transplant Surgery, University Medical Center of the Johannes Gutenberg University Mainz, 55131 Mainz, Germany; jens.mittler@unimedizin-mainz.de; 5 German Center for Cardiovascular Research (DZHK), Partner-Site Rhine-Main, 55131 Mainz, Germany; 6 Division of Cardiovascular Imaging, Department of Radiology and Radiological Science, Medical University of South Carolina, Charleston, SC 29425, USA

**Keywords:** carcinoma, hepatocellular, immunotherapy, diagnostic imaging, treatment outcome, spleen volume, prognosis

## Abstract

**Simple Summary:**

Splenic volume (SV) has been identified as a highly predictive parameter for prognosis in patients with hepatocellular carcinoma (HCC). Moreover, an association between immunotherapy and an increase in SV has been described for various types of cancer. In our cohort of patients with HCC under immunotherapy, SV was a highly predictive factor for overall survival at baseline and initial follow-up. Although a large proportion of patients (76%) showed an SV increase after the initiation of immunotherapy, this additional immuno-modulated SV change was negligible compared to long-standing changes in the splanchnic circulation in our patient cohort.

**Abstract:**

*Background*: An association between immunotherapy and an increase in splenic volume (SV) has been described for various types of cancer. SV is also highly predictive of overall survival (OS) in patients with hepatocellular carcinoma (HCC). We evaluated SV and its changes with regard to their prognostic influence in patients with HCC undergoing immunotherapy. *Methods*: All patients with HCC who received immunotherapy in first or subsequent lines at our tertiary care center between 2016 and 2021 were screened for eligibility. SV was assessed at baseline and follow-up using an AI-based tool for spleen segmentation. Patients were dichotomized into high and low SV based on the median value. *Results*: Fifty patients were included in the analysis. The median SV prior to treatment was 532 mL. The median OS of patients with high and low SV was 5.1 months and 18.1 months, respectively (*p* = 0.01). An increase in SV between treatment initiation and the first follow-up was observed in 28/37 (75.7%) patients with follow-up imaging available. This increase in itself was not prognostic for median OS (7.0 vs. 8.5 months, *p* = 0.73). However, patients with high absolute SV at the first follow-up continued to have impaired survival (4.0 months vs. 30.7 months, *p* = 0.004). *Conclusion*: High SV prior to and during treatment was a significant prognostic factor for impaired outcome. Although a large proportion of patients showed an SV increase after the initiation of immunotherapy, this additional immuno-modulated SV change was negligible compared to long-standing changes in the splanchnic circulation in patients with HCC.

## 1. Introduction

Hepatocellular carcinoma (HCC) is the most common primary liver cancer and one of the leading causes of cancer-related deaths worldwide [1]. Patients suffering from HCC tend to have two underlying diseases that influence their prognosis and treatment outcome; in more than 80% of Western patients, HCC developed in existing liver cirrhosis [2]. Thus, in addition to the tumor burden, survival is heavily influenced by the remaining liver function. Liver cirrhosis itself leads to the development of portal hypertension [3]. Portal hypertension, in turn, is a factor influencing the risk of hepatic decompensation during HCC treatment and is furthermore a prognostic factor for overall survival (OS) [4,5,6]. The reference standard for measuring portal hypertension is direct measurement of the hepatic vein pressure gradient (HVPG) through a transjugular approach [2,4]. However, due to its invasive nature and high effort, HVPG measurement is not routinely performed in the diagnostic evaluation of patients with HCC. Consequently, other clinical parameters, such as low platelet count, the presence of esophageal/gastric varices, and ascites, are considered surrogates in the identification of patients with clinically relevant portal hypertension (CRPH) [7,8,9].

Splenic volume (SV) at baseline and during treatment has also been identified as a surrogate for CRPH in patients with HCC [10]. Furthermore, it is highly relevant for predicting the prognosis in patients with HCC undergoing curative and palliative treatment [11,12,13,14,15]. Novel AI-based methods enable a fully automated assessment of the SV using computed tomography (CT) data [15,16]. Thus, it can be considered a promising imaging biomarker with the potential for full integration into the routine radiology workflow. 

In recent years, the results of the IMbrave150 trial led to changes in the treatment paradigm: Immunotherapy has become a first-line systemic treatment option for patients with advanced HCC and for patients in whom other treatment options have failed [17,18,19,20,21]. Furthermore, several ongoing trials are currently investigating other potential immunotherapeutic agents in various tumor stages [19,22,23]. However, immunotherapy has been linked to systemic reactions and shown to influence several organ systems besides the target [24]. One organ that is affected is the spleen. A change in SV during treatment has been previously reported for patients with melanoma and lung cancer [25,26]. Furthermore, SV has been identified as a risk factor for survival outcomes [26].

No study has yet investigated the role of SV and changes in SV in patients with HCC receiving immunotherapy. Given the high coincidence of concomitant liver cirrhosis and increased SV prior to treatment, the present study aimed to investigate whether additional immuno-modulated changes in SV occur and have a detrimental effect in HCC patients undergoing immunotherapy.

## 2. Materials and Methods

The Ethics Committee of the Medical Association of Rhineland Palatinate, Mainz, Germany, approved this study (permit number 837.199.10). The requirement for informed consent was waived due to the retrospective nature of the study. This report followed the guidelines for reporting observational studies (STROBE) [27].

### 2.1. Patients

**Figure 1:**
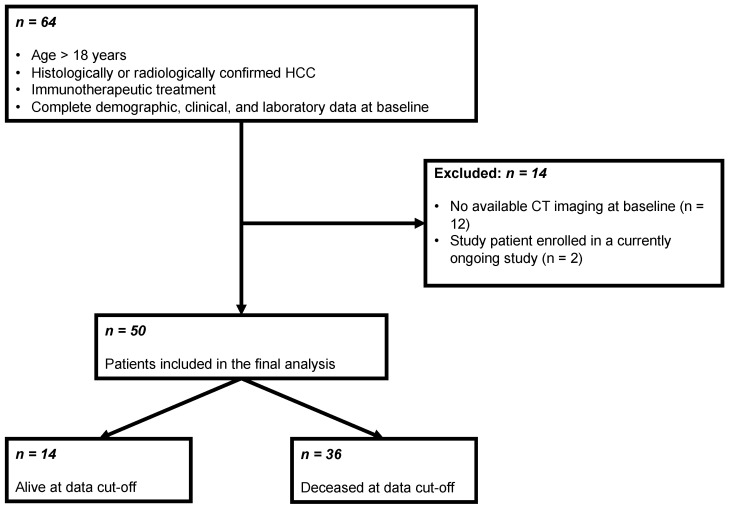
Flowchart of the patient selection process for this study.

This retrospective study included all patients with HCC who presented in our dedicated HCC outpatient clinic between May 2016 and October 2021 for the initiation of immunotherapy. Inclusion criteria were age > 18 years, histological or image-derived HCC diagnosis based on the EASL criteria, immunotherapy as systemic treatment, CT images available prior to immunotherapy, and demographic, clinical, and laboratory data available at initiation of the immunotherapy and during follow-up. Of the scanned 64 patients, 50 (78.1%) patients fulfilled all inclusion criteria (Figure 1).

### 2.2. Diagnosis, Treatment, and Follow-Up

As previously reported, histological or image-derived EASL criteria were used for the diagnosis of HCC [2,28]. The decision to initiate immunotherapy was made by an interdisciplinary tumor board. The board consisted of hepatologists/oncologists, diagnostic and interventional radiologists, visceral surgeons, pathologists, and radiation therapists, who discussed each case prior to the treatment decision. All patients received contrast-enhanced multiphasic CT imaging prior to treatment initiation. Follow-up consisted of clinical examination, blood sampling, and cross-sectional imaging, which was typically repeated every 6 to 12 weeks.

### 2.3. Splenic Volume Assessment

SV was assessed using an established tool for fully automated segmentation and volumetry of the spleen installed at our institution [15]. This algorithm employs the open-source MIScnn library, a convolutional neural network with a U-Net architecture, and has previously been trained for spleen segmentation in patients with HCC undergoing transarterial chemoembolization (TACE) [29]. Detailed information on the features of the network, the settings for training and validation, and the model’s performance can be found in the original publication [15]. The output of the network consisted of graphic overlays, which were reviewed by two independent readers. The quality of the graphic overlays was rated as perfect, acceptable, or poor. Consensus reading was performed in the case of discrepancies (*n* = 2 (4.0%)). Patients with perfect or acceptable SVs were included in the statistical analyses (*n* = 48); patients with a poor grade (*n* = 2) were manually re-segmented to obtain the proper SV for further analyses as reported previously [15]. For manual segmentation, the freely available LIFEx software was used (www.lifexsoft.org) [30]. In a second step, SV was normalized to the body surface area (BSA), which was calculated using the patient’s height and weight.

### 2.4. Statistical Analysis

Statistical analyses and graphic design were performed in R 4.0.3 (A Language and Environment for Statistical Computing, R Foundation for Statistical Computing, http://www.R-project.org; accessed on 31 May 2022). Data distribution of the continuous variables was assessed for normality using the Shapiro–Wilk test. Normally distributed variables were expressed as mean and standard deviation (SD), whereas non-normally distributed variables were expressed as median and interquartile range (IQR). Categorical and binary baseline parameters were reported as absolute numbers and percentages. Categorical parameters were compared using Fisher’s exact test and continuous parameters using the Student’s t-test in case of normal distribution and the Mann–Whitney test in case of non-normal distribution. Survival analyses and creation of the Kaplan–Meier curves were performed using the packages “survminer” and “survival” (https://cran.r-project.org/package=survminer, https://CRAN.R-project.org/package=survival, accessed on 31 May 2022). For all patients, OS and progression-free survival (PFS) were calculated from the initiation of treatment. In addition, for patients with available follow-up imaging, OS was calculated from the first follow-up. Log-rank testing was used to compare survival times. Cox proportional hazards regression models assessing hazard ratios (HRs) and corresponding 95% confidence intervals (CIs) were used to determine the effect of the risk stratification. Significance was set at *p* < 0.05.

## 3. Results

### 3.1. Baseline Characteristics

A total of 50 patients, 40 males (80.0%) and 10 females (20.0%), with a median age of 68 years (IQR 62–73 years), were included in the final analysis. For the 37 (74.0%) patients with follow-up CT available, the median time between treatment initiation and follow-up imaging was 85 days (range 68–100 days). Baseline characteristics are provided in Table 1.

### 3.2. Increase in Splenic Volume after Initiation of Immunotherapy

**Figure 2:**
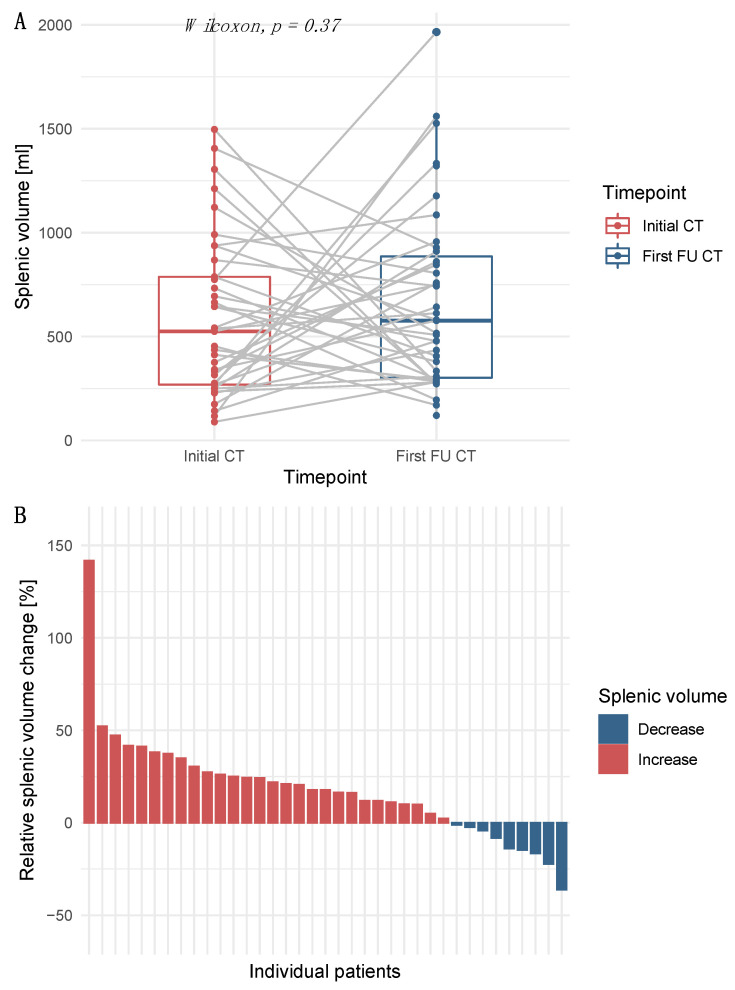
Splenic volume (SV) at baseline and during treatment with immunotherapy agents. (**A**) Boxplots of the SV at baseline and during follow-up. (**B**) Relative individual changes in SV between baseline and first follow-up.

The median SV for all patients was 531.8 mL (IQR 270.4–784.4 mL) and the SV to BSA ratio was 261.9 mL/m² (IQR 148.1–397.8 mL/m²). For the 37 (74.0%) patients with CT follow-up imaging available, the median SV at baseline was 524.8 mL (IQR 268.7–784.8 mL) and the SV to BSA ratio was 273.0 mL/m² (IQR 163.3–414.8 mL/m²). The median SV at the first follow-up was 576.9 mL (IQR 307.6–860.7 mL) for these patients (*p* = 0.37; Figure 2A). An increase in the SV was observed in 28 (75.7%) patients, whereas 9 (24.3%) patients had a decrease in SV during early treatment (Figure 2B). The median change in SV was 17.8% (IQR 2.2–27.3%; range-36.1–141.7%).

For the following analyses, patients were dichotomized into high and low SV based on the median SV to BSA ratio of the patient cohort. According to this stratification, among initial and follow-up imaging, a change from the low to high SV group was observed in only 2 (5.4%) patients, whereas 35 (94.6%) patients remained in their initial group.

### 3.3. Correlation of Splenic Volume with Parameters of Liver Function, but Not with Tumor Burden

Patients with high SV had significantly lower albumin levels, higher bilirubin levels, and fewer thrombocytes. No significant differences were observed regarding the INR, the sum of the target lesions, the presence of portal vein infiltration, and the presence of distant metastasis (Table 2).

### 3.4. Independence of Splenic Volume and Radiological Response

**Figure 3:**
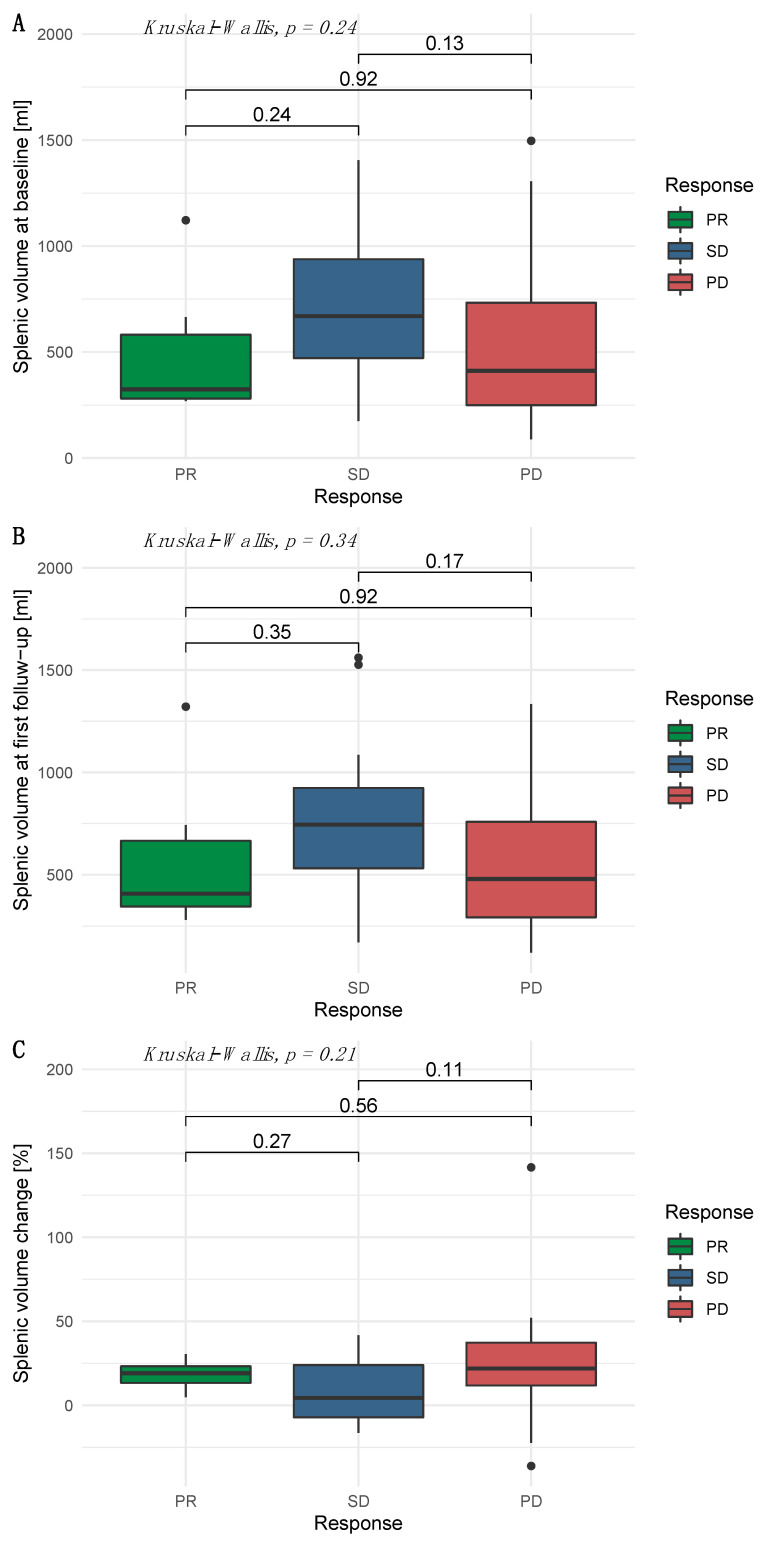
Splenic volume among the various response categories. (**A**) Baseline, (**B**) follow-up, and (**C**) relative change.

For patients with available follow-up imaging, radiological response was assessed according to mRECIST. The baseline SV of patients with a partial response, stable disease, and progressive disease was 324 mL (IQR 280–581 mL), 670 mL (IQR 471–938 mL), and 412 mL (IQR 249–733 mL), respectively. The follow-up SV in patients with a partial response, stable disease, and progressive disease was 407 mL (IQR 345–666 mL), 744 mL (IQR 531–923 mL), and 479 mL (IQR 293–758 mL), respectively. The median relative change in SV in patients with a partial response, stable disease, and progressive disease between initial imaging and follow-up was 19.1% (IQR 13.3–23.1%), 4.4% (IQR −7.2–24.0%), and 21.9% (IQR 11.9–37.3%), respectively (Figure 3).

### 3.5. Significant Impact of High Splenic Volume at Treatment Initiation and during Follow-Up on Overall Survival

**Figure 4:**
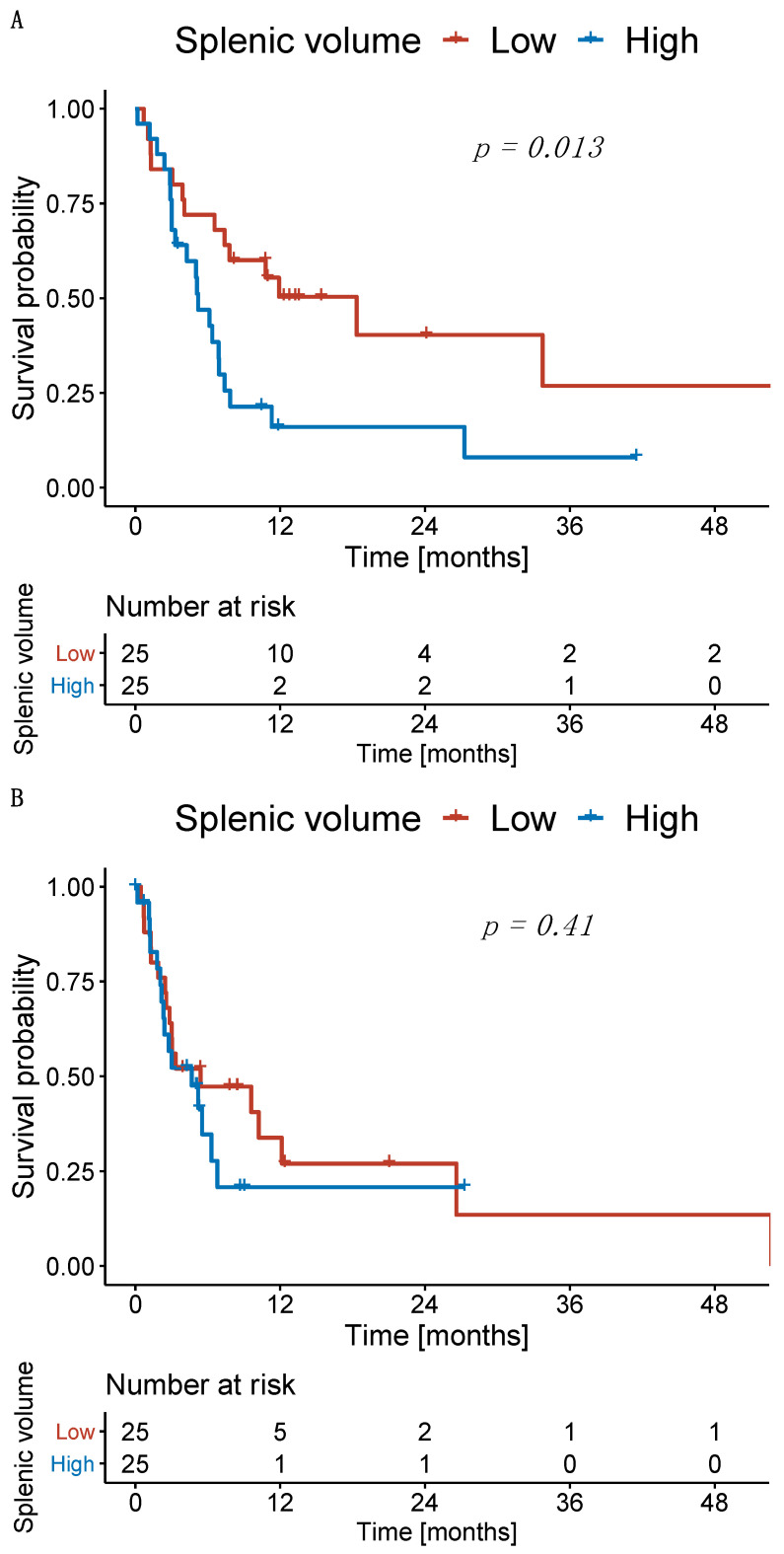
Kaplan–Meier curves for patients with low and high splenic volume. (**A**) Overall survival and (**B**) progression-free survival.

The median OS of patients with high SV at baseline was 5.1 months, whereas patients with a low SV had a median OS of 18.1 months (*p* = 0.013; Figure 4A). The PFS in patients with high SV at baseline was 4.6 months, whereas patients with a low SV had a median PFS of 5.3 months (*p* = 0.410; Figure 4B).

**Figure 5:**
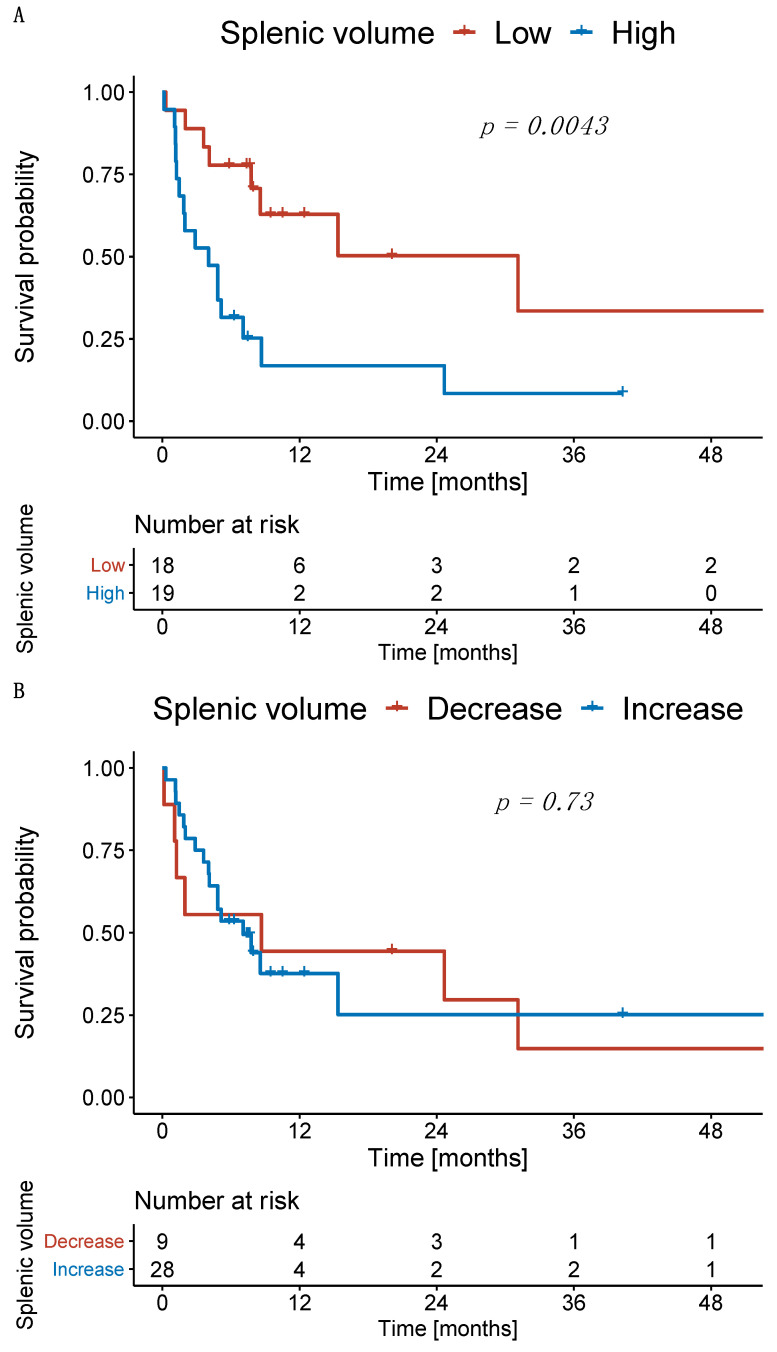
Kaplan–Meier curves for overall survival. (**A**) Patients stratified according to the splenic volume at first follow-up and (**B**) according to the relative change compared to baseline.

Subsequently, we investigated the survival of patients with high and low SV at the first follow-up. Patients with high and low SV at the first follow-up had a median OS of 4.0 months and 30.7 months (*p* = 0.004), respectively (Figure 5A). Patients with an increase in SV from baseline to first follow-up had a median OS of 7.0 months, whereas patients with a decrease in SV had a median OS of 8.5 months (*p* = 0.730; Figure 5B).

## 4. Discussion

In this study, we investigated the role of SV and changes in SV with regard to survival outcomes after the initiation of immunotherapy in patients with HCC. Baseline SV was a significant prognostic factor for OS. During early follow-up, the majority of patients had an increase in SV after the initiation of treatment. However, only the absolute SV at the first follow-up remained a significant prognostic factor, and there was no significant survival difference in patients with an increase or decrease in SV.

Our results are in line with previous reports on the changes in SV in patients treated with immunotherapy for other cancer entities [25,26]. Susok et al. investigated the changes in SV during treatment initiation in 49 patients with stage III and IV melanoma [25]. The authors reported a significant increase in the SV after 3 months of follow-up and particularly with the use of anti-CTLA-4 and anti-CTLA-4/anti-PD-1 regimens [25]. However, they did not identify a significant relationship with other clinical parameters. In our study, approximately three-fourths of the patients showed an increase in SV during follow-up, and the median SV increased from 525 to 577 mL, though this increase was not significant. 

The median SV change of approximately 18% in our cohort was higher than previously reported for patients with non-small-cell lung cancer undergoing immunotherapy [26]. In their study, Galland et al. reported an increase in 63.5% of patients and a median change of 4.4%. Similar to our results, PFS was not associated with the SV, and the authors reported a significant influence of the baseline SV and the SV during treatment on OS. Unfortunately, the authors did not provide the median absolute SV at treatment initiation and during follow-up. However, the cut-offs used for patient stratification indicate a large difference in the median SV in our patients [26] due to the high proportion of patients with chronic liver disease in our cohort and the associated increase in SV due to increased pressure in the splanchnic circulation [3]. In contrast to our results, Galland et al. postulated that the increase in SV during treatment was significantly associated with impaired survival [26]. In our study, log-rank testing did not show a significant difference in the survival distribution of patients with an increase or decrease in SV during treatment. Moreover, the change in SV under immunotherapy resulted in a change from the low to high SV group in only two (5%) patients. Therefore, the short-term immuno-modulated increase in SV seems to be less important than the pre-existing increase in SV induced by long-standing changes to the splanchnic circulation. Thus, the etiology of changes in SV seems to play a role in investigating correlations between SV and patient outcomes. This is underlined by the significant association between SV and liver function in our study.

In patients with HCC, the baseline SV has been identified as a relevant prognostic factor in various treatment modalities [11,12,13,14,15]. Our results confirm the importance of SV during initial patient evaluation. However, manual spleen segmentation is time-consuming and has a high risk of inter-rater variance [31]. Thus, AI-based solutions for automated SV assessment have the potential to facilitate and standardize this task and enable easy integration into radiological routine. The feasibility of such concepts was reported previously for patients with liver cirrhosis and HCC [15,16]. In this study, we used an algorithm that we had previously trained for patients with HCC undergoing TACE and showed high accuracy in both training and validation [15]. In our study, the algorithm showed sufficient segmentation in 96%, confirming the results of the original study [15]. The present study highlights the easy integration of SV assessment into the routine workflow, together with the high prognostic importance of SV for patients with HCC undergoing immunotherapy. Thus, SV assessment should be contemplated in the diagnostic work-up and for estimating the prognosis in these patients prior to initiating treatment.

The results of this study must be considered in light of several limitations. First, this study was conducted in a retrospective manner and included a limited number of patients. However, this dataset was well-investigated and only patients with complete clinical, laboratory, and imaging data were included. No imputation of missing values was performed. Second, we decided to include patients treated with various immunotherapeutic agents to validate the role of SV in a real-life clinical setting. We did not perform subgroup analysis on each immunotherapy agent due to the small number of patients in each subgroup. However, future studies should validate SV as a novel prognostic factor for various immunotherapy agents and treatment lines.

## 5. Conclusions

In patients with HCC undergoing immunotherapy, high SV prior to and during treatment was a significant prognostic factor for impaired survival. Although a large proportion of HCC patients in our cohort had an SV increase after the initiation of immunotherapy, this increase during treatment did not negatively affect OS per se. Thus, additional immuno-modulated changes in SV were negligible compared to long-standing changes in the splanchnic circulation in patients with HCC.

## Figures and Tables

**Table 1:** Baseline characteristics.

Parameter	All Patients (*n* = 50)
Age, years *	67.2 (9.0)
Sex ***	
Female	10 (20.0)
Male	40 (80.0)
Etiology of cirrhosis ***	
Alcohol	19 (38.0)
Viral	7 (14.0)
Other	11 (22.0)
No cirrhosis	13 (26.0)
**Child**–Pugh stage ***	
A	25 (50.0)
B	10 (20.0)
C	2 (4.0)
No cirrhosis	13 (26.0)
ECOG ***	
≤1	47 (94.0)
2	3 (6.0)
BCLC stage ***	
B	5 (10.0)
C	42 (84.0)
D	3 (6.0)
Portal vein invasion ***	
Yes	26 (52.0)
No	24 (48.0)
Distant metastasis ***	
Yes	25 (50.0)
No	25 (50.0)
Focality of the liver lesions ***	
Unifocal	11 (22.0)
Multifocal	39 (78.0)
Sum of the target lesion sizes, mm **	83 (51–135)
AFP, ng/mL **	277 (16–4485)
Albumin, g/L *	30.4 (5.4)
Bilirubin, mg/dL **	1.5 (0.7–2.3)
INR **	1.2 (1.1–1.3)
Creatinine, mg/dL **	0.9 (0.7–1.1)
Thrombocytes, per nL **	139 (94–260)
Immunotherapy agent ***	
Atezolizumab + bevazizumab	29 (58.0)
Pembrolizumab	11 (22.0)
Nivolumab	10 (20.0)
Line of systemic treatment ***	
First	29 (58.0)
Second	11 (22.0)
Third	10 (20.0)
Previous therapy ***	
Yes	42 (84.0)
No	8 (16.0)
Subsequent therapy ***	
Yes	13 (26.0)
No	37 (64.0)

Values are given as * mean (SD), ** median (IQR) or *** n (%). AFP, alpha-fetoprotein; INR, International Normalized Ratio.

**Table 2:** Comparison of liver function- and tumor burden-related parameters in patients with low and high splenic volume (SV).

Parameter	Low SV (*n* = 25)	High SV (*n* = 25)	*p*-Value
Liver function			
Albumin, g/L *	32.2 (5.9)	28.5 (4.21)	0.014
Bilirubin, mg/dL **	0.8 (0.6–1.6)	2.1 (1.5–2.7)	<0.001
Thrombocytes, per nL **	224 (138–315)	101 (75–139)	<0.001
INR **	1.1 (1.1–1.3)	1.2 (1.1–1.4)	0.190
Tumor burden			
Sum of the target lesions, mm **	76 (50–122)	88 (51–156)	0.663
Presence of portal vein infiltration ***	13 (52.0)	13 (52.0)	1.000
Presence of distant metastasis ***	16 (64.0)	9 (36.0)	0.089

Values are given as * mean (SD), ** median (IQR) or *** n (%).

## Data Availability

Data cannot be shared publicly because of institutional and national data policy restrictions imposed by the Ethics Committee of the Medical Association of Rhineland Palatinate, Mainz, Germany, since the data contain potentially identifying patient information. Data are available upon request for researchers who meet the criteria for access to confidential data.
